# Immunization of Aged Pigs with Attenuated Pseudorabies Virus Vaccine Combined with CpG Oligodeoxynucleotide Restores Defective Th1 Immune Responses

**DOI:** 10.1371/journal.pone.0065536

**Published:** 2013-06-13

**Authors:** Feiping Ming, Jun Yang, Pinpin Chu, Miaopeng Ma, Juqing Shi, Haiming Cai, Chaoyuan Huang, Huazhou Li, Zhenggu Jiang, Houguang Wang, Weifang Wang, Shuiqing Zhang, Linghua Zhang

**Affiliations:** 1 College of Life Sciences, South China Agricultural University, Guangzhou, Guangdong, China; 2 Swine Seed Breeding Center of Guangzhou, Guangzhou, China; 3 Guangdong Provincial Key Laboratory of Protein Function and Regulation in Agricultural Organisms, South China Agricultural University, Guangzhou, Guangdong, China; The Ohio State University, United States of America

## Abstract

**Background and Aims:**

Attempts to immunize aged subjects often result in the failure to elicit a protective immune response. Murine model studies have shown that oligonucleotides containing CpG motifs (CpG ODN) can stimulate immune system in aged mice as effectively as in young mice. Since many physiological and pathophysiological data of pigs can be transferred to humans, research in pigs is important to confirm murine data. Here we investigated whether immunization of aged pig model with attenuated pseudorabies virus vaccine (PRV vaccine) formulated with CpG ODN could promote a successful development of immune responses that were comparable to those induced in young pigs in a similar manner.

**Methodology:**

Young and aged pigs were immunized IM with PRV vaccine alone, or in combination with CpG ODN respectively. At days 3, 7, 14 post immunization sera were assayed by ELISA for IgG titres, at day 7 for IgG1 and IgG2 subtypes titres. All blood samples collected in evacuated test tubes with K-EDTA at day 7 were analyzed for flow cytometer assay. Blood samples at day 7 collected in evacuated test tubes with heparin were analysed for antigen-specific cytokines production and peripheral blood mononuclear cells (PBMCs) proliferative responses.

**Results:**

CpG ODN could enhance Th1 responses (PRV-specific IgG2/IgG1 ratio, proliferative responses, Th1 cytokines production) when used as an adjuvant for the vaccination of aged pigs, which were correlated with enhanced CD4+ T cells percentage, decreased CD4+CD8+CD45RO+ T cells percentage and improved PRV-specific CD4+ T cells activation.

**Conclusions:**

Our results demonstrate a utility for CpG ODN, as a safe vaccine adjuvant for promoting effective systemic immune responses in aged pig model. This agent could have important clinical uses in overcoming some of age-associated depressions in immune function that occur in response to vaccination.

## Introduction

Many studies indicate that nearly every component of the immune system undergoes dramatic age-associated remodeling, leading to changes that include enhanced as well as diminished functions [1], [2]. Consistent with consequences of the senescent immune system, aged individuals demonstrate a reduced capacity to be successfully vaccinated [3]. Attempts to immunize elderly humans or aged experimental animals against infectious agents or bacterial toxins, oftentimes result in the failure to elicit a protective immune response [3]–[5]. Several approaches have been pursued in an attempt to increase the immunogenicity of vaccines for use in elderly populations. In general such attempts have failed to induce a reliable and durable enhancement of antibody responses. One strategy for improving the responsiveness of aged individuals to primary immunization would be to develop vaccines containing adjuvants capable of enhancing the function of immune cell types associated with the initiation and elicitation of protective immunity.

The various biological functions of oligonucleotides containing CpG motifs (CpG ODN) have received extensive attentions, especially as vaccine adjuvant against infections in young mice [6]–[7]. Recently, several reports had demonstrated that subcutaneously (s.c.) administered CpG ODN stimulated the immune system in aged mice as effectively as in young mice [8]–[9]. To confirm murine data and gain more insight into the immmunostimulatory activity of CpG ODN in aged organism, we chose aged pig as an animal model for the following reasons: 1) Pigs may be better than mice in which to model human genetic diseases because their anatomy, biochemistry, physiology, size, and genetics are more similar to those of human, such as cystic fibrosis, atherosclerosis, cerebral thrombosis, rheumatoid arthritis, and wound healing [10]. 2) many physiological and pathophysiological data which can be transferred to human and research in other species, are important to confirm murine data; 3) the main lymphoid cell populations of pig are consistent with those of other vertebrates, especially those of human; and 4) the immune system of pig is of interest for veterinary as well as human biomedical research because pig is increasingly recognized as a donor for xeno-transplantation [11].

It was demonstrated in our laboratory that CpG ODN delivered by systemic or mucosal immunization routes could effectively activate systemic and mucosal immune responses to vaccine [12]–[14] in neonatal and weaned piglets. However, there is little or no information about ability of CpG-ODN to stimulate the senescent immune system in aged pigs. Pseudorabies virus, the causative agent of Aujeszky’s disease, is a neurotropic alphaherpesvirus that produces fatal encephalitis in newborn pigs and a milder syndrome in older animals [15]. We [4], [12], [14] had reported good immune responses induced by a attenuated pseudorabies virus (PRV) vaccine in seronegative young and aged pigs, the aim of this study was therefore to evaluate the effectiveness of PRV vaccine/CpG-ODN intramuscular (IM) administration to induce an PRV-specific immune responses in aged pigs. Herein we showed that CpG ODN formulated with PRV vaccine could restore the impaired Th1 responses in aged pigs, which were associated with enhanced PRV-specific CD4+ T cells priming. Our findings suggest that the incorporation of CpG ODN into vaccine formulations provided to the aged could prove useful in the development of more effective vaccines for the elderly.

## Materials and Methods

### Reagents

Culture media (RPMI-1640), Concanavalin A (ConA), Tetramethyl benzidine (TMB), phenyltetrazolium bromide (MTT), and Bovine serum albumin (BSA) halothane were purchased from Sigma. Fetal bovine serum was purchased from Hyclone. B-class CpG-ODN (TCGTCGTTGTCGTTTTGTCGTT) synthesized in the TaKaRa Biotech Co. [16], [17], were used in this study. All ODN possessed a phosphorothioate backbone. ODN was resuspended in phosphate buffer saline (PBS) at a concentration of 2 mg/mL, and stored at −20°C. Attenuated pseudorabies vaccine was purchased from HaErBing Veterinary Institute, Chinese Academy of Science, containing pseudorabies virus Bartha-K61 strain at least 5000 TCID50/ml, 10 wk age group was inoculated with 1 ml, 5.5 year age group with 2 ml per pig. Pseudorabies virus antibody test kit was purchased from the IDEXX, German. Evacuated test tubes with heparin were purchased from HuNai Biotech Co. LTD, China. 96-well plates were purchased from Corning, USA. Mouse anti porcine IgG2, IgG1 and horseradish peroxidase-labeled mouse anti porcine IgG were purchased from Serotec, Kidlington, UK. Horseradish peroxidase-labeled goat anti mouse IgG was purchased from Southern Biotechnology Associates, Inc. Swine IL-4, IL-5, IFN-γ and IL-12 immunoassay kits were purchased from R&D, Nasdaq, USA.

Evacuated test tubes with heparin were purchased from HuNai Biotech Co. LTD, Hunan, CN. Antibody for flow cytometry analysis: anti-pig CD3- SPRD, anti-pig CD4a- PE, anti-pig CD8a-R- FITC, anti-pig CD45RA and anti-pig CD45RO were respectively from Serotec, Kidlington, UK and Abcam, Cambridge, UK. Secondary antibodiy (rat anti-mouse IgG2a–APC) was from BD Biosciences, California, USA. Ficoll- Hypaque PLUS was from Pharmacia Biotech Corp., Uppsala, Sweden.

### Immunization of Animals

All studies were approved by the Guangdong Science and Technology Department. Landrace×Yorkshire×Durok Pigs (Swine Seed Breeding Center of Guangzhou) of different ages were used in this study, and 5.5 year-old pigs were seen as aged pigs 4,18. The conventional pigs were kept under standard farming conditions. The age groups, breeding conditions, number of animals and studied animals are given in Table 1.

**Table 1:** Pigs used in the study.

Age groups	Breeding conditions	Animal numbers
10 wk	Conventional	10
10 wk-CpG	Conventional	10
5.5 year	Conventional	10
5.5 year-CpG	Conventional	10

To evaluate the immune-enhancing effects of CpG ODN in aged pigs, young and aged pigs were immunized IM with PRV vaccine (containing pseudorabies virus Bartha-K61 strain at least 5000TCID50/ml, 10 wk age group was inoculated with 1 ml, 5.5 year age groups with 2 ml per pig) alone, or in combination with 350 µg (for 10 wk pig) or 1 mg (for 5.5 year pig) CpG ODN respectively. The dosages of CpG ODN used corresponded to those previous data by Zhang et al. [19]. At days 3, 7, 14 post immunization sera were collected and stored at −20°C until assayed by ELISA for IgG titres, at day 7 for IgG1 and IgG2 subtypes titres. All blood samples collected in evacuated test tubes with K-EDTA at day 7 were analyzed for flow cytometer assay. Blood samples at day 7 collected in evacuated test tubes with heparin were analysed for antigen-specific cytokines production and peripheral blood mononuclear cells (PBMCs) proliferative responses. All protocols were carried out in accordance with China Council of Animal Care Guide to the Care and Use of Experimental Animals.

### Analyses of Antibody Titres

Antigen-specific IgG and IgG1/IgG2 subtypes from sera were determined by end-point ELISA using methods previously described [12], [14], [20].

### Cell Preparation

PBMCs were prepared by density gradient centrifugation of heparized peripheral blood samples obtained from piglets as previously reported [21]. Briefly, a volume of peripheral blood was diluted with an equal volume of D-PBS and layered on Ficoll-Hypaque PLUS. After centrifugation at 350×g at room temperature for 20 min, the layer containing PBMC fraction was obtained and washed once with medium (RPMI 1640 containing 10% heat inactivated pooled fetal bovine serum, antibiotics (penicillin 100 U/mL and streptomycin 0.1 mg/mL and L-glutamine 2 mM). Red blood cells were removed after lysis in ammoniumchloride (0.8%, w/v) and the remaining cells were washed twice in cold RPMI-5% FCS. And then cells were resuspended in medium at a cell concentration of 1×10^6^ cells/mL.

### Cytokines Assay

PRV vaccine was diluted to concentration (1 mg/ml) in RPMI 1640, which was used as the swine PRV antigen. IFN-γ and IL-6 production was assessed by culturing PBMCs (1×10^6^ cells/mL) in triplicate with PRV antigen (10 µg/mL). Control stimuli included RPMI 1640 medium alone or ConA at 5 µg/mL. Supernatants were harvested after 72 h at 37°C with 5% CO_2_ atmosphere, filtered and stored at −20°C until assayed. The presence of porcine IFN-γ, IL-12 p40, IL-5 and IL-4 in porcine PBMC culture supernatants were determined by commercial swine immunoassay kits according to the manufacturer’s directions. Supplied standards were used to generate a standard curve. The detection limits of assays were 2.0 pg/mL for IFN-γ, IL-12 p40 and IL-5, 4.0 pg/mL for IL-4.

### PRV-specific T Cell Assay

Purified PBMCs were suspended in complete RPMI-1640 without the phenol red indicator to achieve 2.5×10^6^ cells/mL. Untreated cells (without addition of PRV antigen) were cultured in RPMI-1640 medium with 10% fetal bovine serum and without the phenol red indicator, 0.15% sodium bicarbonate and 1% antibiotic/antimycotic. Treated cells were cultured under the same conditions, but with the addition of PRV antigen (at a final concentration of 10 µg/mL) in complete RPMI-1640 without the phenol red indicator. The primary incubation of these cells was at 37°C with 5% CO_2_ for 24 h, followed by addition of 10 µL MTT (5 mg/mL) per well and a secondary incubation at 37°C with 5% CO_2_ for another 1 h. Centrifuge microplates and remove unreacted MTT, and then add 100 µL dimethyl sulfoxide (DMSO) per well to solubilize formazan, incubating 5 min with shaking. The absorbance plate was readed using a 570 nm filter within minutes. The geometric means and standard deviations for triplicate sets of samples were calculated. Lymphocyte proliferation is expressed as a stimulation index (SI), which was defined as the mean of PRV-treated data divided by the mean of the untreated control [22]–[23].

### Flow Cytometer Assay

The PBMCs isolated at day 7 post immunization were placed into culture with PRV antigen for 48 h, and then were analyzed for flow cytometer assay. Flow cytometric analyses were performed on blood samples and PRV-stimulated PBMCs as described by our experimental procedure [24]–[26]. Samples collected 3 pools in each group were used for CD3/CD4/CD8/CD45RO and CD3/CD4/CD8/CD45RA four-color cytofluorometric analyses. Isotypes matched antigen-irrelevant control monoclonal antibodies were used in each staining as negative controls to set the quadrant markers for the bivariate dot plots. At least 10000 cells were analyzed per sample. Stained cells were analyzed by cytofluorometry (FACSCalibur, dual-laser, Becton & Dickinson, USA), and the data were analyzed with Cellquest V3.3 software. Results were reported as percentage of cell populations expressing the markers of interest among gated cells.

### Statistical Analysis

Data were analyzed using the statistical software program Systat 10 (SPSS 10). Distribution of data was determined using descriptive statistics. Data which were not normally distributed were transformed by ranking. Differences in ELISA titers were investigated using one-way analysis of variance (ANOVA) performed on the rank. Means of the rank were compared using Tukey’s multiple comparison tests. A *p*-value of <0.05 was considered significant.

## Results

### CpG ODN Restores Antibody Responses following IM Delivery with PRV in Aged Pigs to Young Pigs

Our recent studies have indicated that young pigs given vaccine formulations containing CpG ODN as the adjuvant responded by generating potent humoral and cellular immune responses 12,16. To investigate whether vaccines containing CpG ODN might enhance the immunization efficacy in aged animals, groups of young pigs and aged pigs were immunized IM with PRV vaccine in the presence or absence of CpG ODN. PRV-specific IgG in sera in different time-points were measured.

Young control pigs that were immunized with PRV alone were capable of mounting good anti-PRV antibody responses, while the aged pigs responded poorly to vaccination with PRV (Table 2). This finding was consistent with our previous studies reporting that aged pigs had a decreased capacity to respond effectively with protein antigens [4]. However, when young and aged animals were immunized with a PRV vaccine formulation containing CpG ODN, the levels of anti-PRV antibodies present in collected serum samples were increased by approximately 3-fold in the young pigs (at day 14) and by 3-fold (at day 14) in aged mice, over the levels of antibodies present in the serum of young or aged pigs immunized with PRV alone (Table 2). Given these data, it is clear that systemic humoral immune responses can be effectively induced in aged animals when CpG ODN was used as an adjuvant in PRV vaccine formulation.

**Table 2:** The titres for PRV-specific serum antibodies following IM delilvery in different age groups*.

Groups	Days post immunization			
	0	3	7	14
10 wk	–	1073±241^a^	2115±107^a^	1874±113^a^
10 wk-CpG	–	3443±547^c^	6217±753^b^	7071±811^c^
5.5 year	–	304±87^b^	1124±103^a^	876±183^b^
5.5 year-CpG	–	836±201^a^	2354±343^a^	2346±467^a^

* Young and aged pigs were immunized IM with PRV vaccine alone, or in combination with CpG ODN respectively. Each data represents the group mean +/− SEM for titers of anti-PRV antibodies as determined in triplicate by end-point dilution ELISA assay. Different letter expressed significant difference between groups *p*<0.05, i.e. the titres in the groups marked with a or b were significant lower than the groups with c, “–” means no detectable IgG antibodies.

Although, there is precedence for CpG ODNs being potent Th1-like adjuvants when delivered via the parenteral (intramuscular and subcutaneous) routes [8] in aged mice, these effects have not been investigated in aged pigs. Therefore, we studied the capacity of CpG/PRV delivered by IM route to elicit Th1 versus Th2 responses on day 7 post immunization. Evaluation of serum for IgG antibody isotypes showed that in the serum of young pigs vaccinated with PRV vaccine, IgG1 was the predominant isotype and low levels of IgG2 were detected, resulting in an IgG2/IgG1 ratio of 0.41 (Table 3). In aged pigs, IgG2 levels were much lower than young pigs, which gave an IgG2/IgG1 ratio of 0.17. However, a significant increase in the level of IgG2 was observed in the 10 wk-CpG (yong pigs) and 5.5 year-CpG (aged pigs) groups. As seen in Table 3, the addition of CpG ODN could enhance IgG2/IgG1 ratio for both young and aged pigs. This suggested an increase in the helper T cell responses of Th1 type induced by CpG ODN.

**Table 3:** The antigen-specific serum IgG1 and IgG2 isotype titre following IM delilvery on day 7 post immunization*.

Groups	IgG1	IgG2	IgG2/IgG1
10 wk	1542±334^a^	632±122^a^	0.41
10 wk-CpG	2415±647^a^	2987±824^c^	1.24
5.5 year	621±131^b^	108±22^b^	0.17
5.5 year-CpG	1154±208^a^	772±164^a^	0.67

* Young and aged pigs were immunized IM with PRV vaccine alone, or in combination with CpG ODN respectively. Each data represents the group mean +/− SEM for IgG1 and IgG2 isotype titres as determined in triplicate by end-point dilution ELISA assay. Different letter expressed significant difference between groups *p*<0.05, i.e. the titres in the groups marked with b were significant lower than the groups with a, the titres in the groups marked with c were significant higher than the groups with a and b.

### CpG ODN used as a Vaccine Adjuvant in Aged Pigs Enhances Systemic PRV-Specific PBMCs Responses

**Figure 1:**
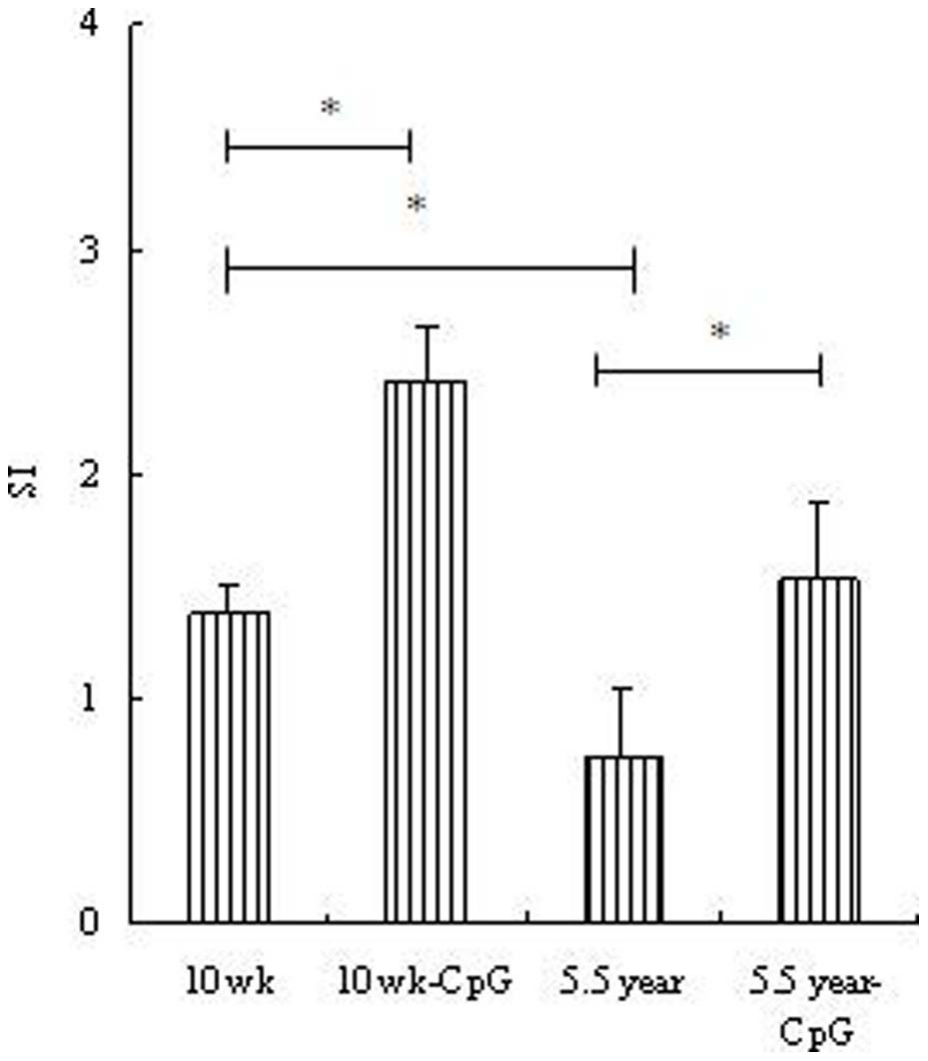
Systemic PRV-specific PBMCs responses. **Legend:** PBMCs were from young (10 wk) and aged (5.5 y) pigs vaccinated with PRV-CpG vaccine or PRV alone at day 7 post immunization.PBMCs were cultured in the presence of PRV antigen for 24 h, followed by addition of MTT, as described under Section PRV-specific T cell assay. ConA served as a positive antigen, i.e. positive control. Each bar represents the group mean +/− SEM of SI determined in triplicate. *p<0.05 indicates significant difference between groups.

Aging mediated PRV-specific lymphocyte proliferation was measured by MTT colorimetric assay method. Representative results of a proliferation study using purified PBMCs of various groups were shown in Fig. 1. Compared with background OD (0.201±0.131) and positive control (SI = 2.47±0.64), aged pigs (5.5 year age) immunized with PRV vaccine had induced significantly weak proliferative responses (*p*<0.05) when compared with young pigs (10 wk age). Similar to the antibodies responses, CpG formulation could also effectively enhance the proliferative responses in both young and aged pigs. The proliferative levels of 5.5 year-CpG group were significnatly higer than 5.5 year (no CpG) group, and were comparable with that of 10 wk group (immunized with PRV alone).

### CpG ODN Enhances Th1 Cytokine Responses in Aged Pigs Induced by IM Deliveries with PRV

**Figure 2:**
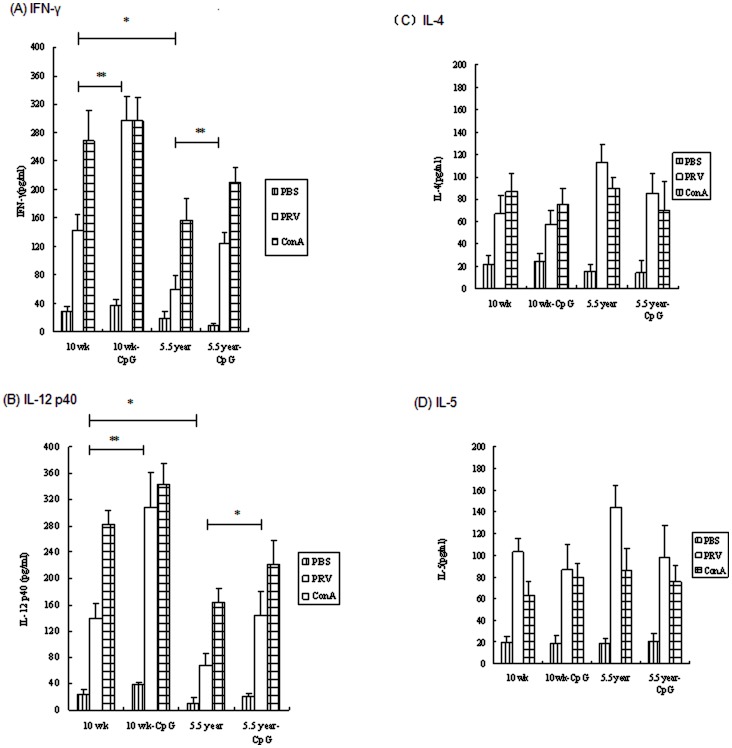
Production of PRV-specific (A) IFN-γ, (B) IL-12 p40, (C) IL-4, and (D) IL-5 in porcine PBMC culture supernatants. Legend: PBMCs were from young (10 wk) and aged (5.5 y) pigs vaccinated with PRV-CpG vaccine or PRV alone at day 7 post immunization. PBMCs treated by ConA (positive antigen), were used as PC, PBMCs treated by medium as NC. Concentrations of cytokines were determined by ELISA as described under Section Cytokines assay. Each bar represents the group mean +/− SEM of cytokines levels determined in triplicate. *p<0.05 and **p<0.01 indicate significant difference between groups.

To further characterize the Th-biased immune responses induced by CpG ODN in aged pigs, PRV-specific IFN-γ, IL-12 p40, IL-4 and IL-5 production by PRV-treated PBMCs from immunized pigs was examined. As expected, Th2 response is of particular concern for aged pigs since IL-4 and IL-5 were both higher in 5.5 year group (aged pigs), compared with 10 wk group (young pigs) which immunized PRV only. Significantly lower Th1 cytokines (IFN-γ and IL-12 p40) were detected in 5.5 year age group compared with 10 wk age group (*p*<0.05) (Fig. 2 A–D). However, CpG formulation could restore the PRV-specific Th1-cytokine responses in aged pigs to young pigs levels (CpG treatment could significantly enhance the IFN-γ and IL-12 p40 levels in aged pigs and young pigs), which further indicated development towards Th1-biased responses in CpG-PRV immunized aged pigs.

### Incorporation of CpG ODN into Vaccine Formulations Changes Phenotype of PBMCs from Aged Pigs

**Figure 3:**
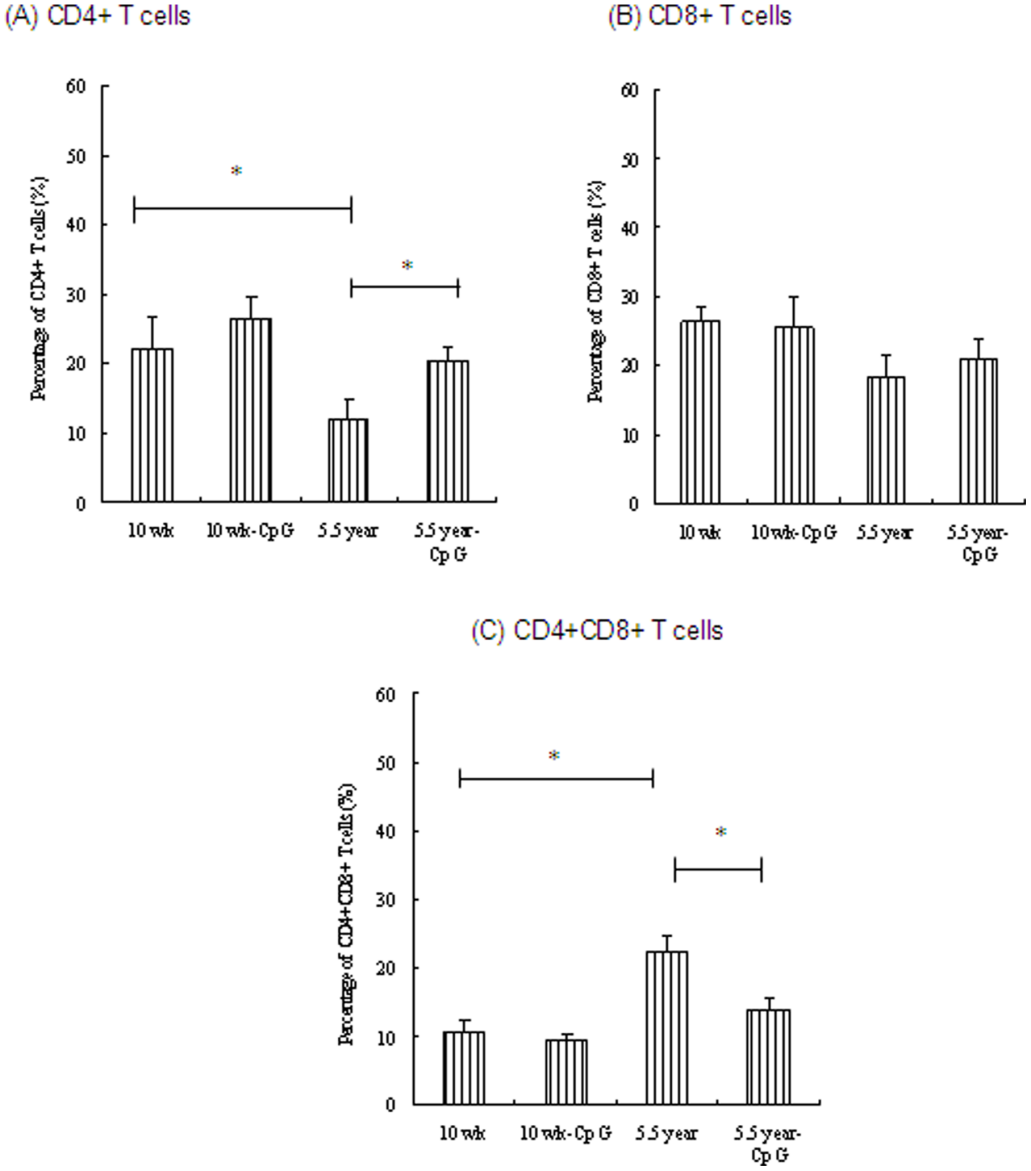
Flow cytometric characterization of CD3+ T cells ((A) CD4+ T cells, (B) CD8+ T cells, (C) CD4+CD8+ T cells) in PBMCs of young (10 wk) and aged (5.5 y) pigs vaccinated with PRV-CpG vaccine or PRV alone. Legend: Samples collected 3 pools in each group were used for CD3/CD4/CD8/CD45RO and CD3/CD4/CD8/CD45RA cytofluorometric analyses. Data were presented as the percentage of cells positive for each surface antigen, mean percentage of leukocyte subsets +/− SEM is shown. Asterisk (*) indicates significant difference between groups (*p*<0.05).

**Figure 4:**
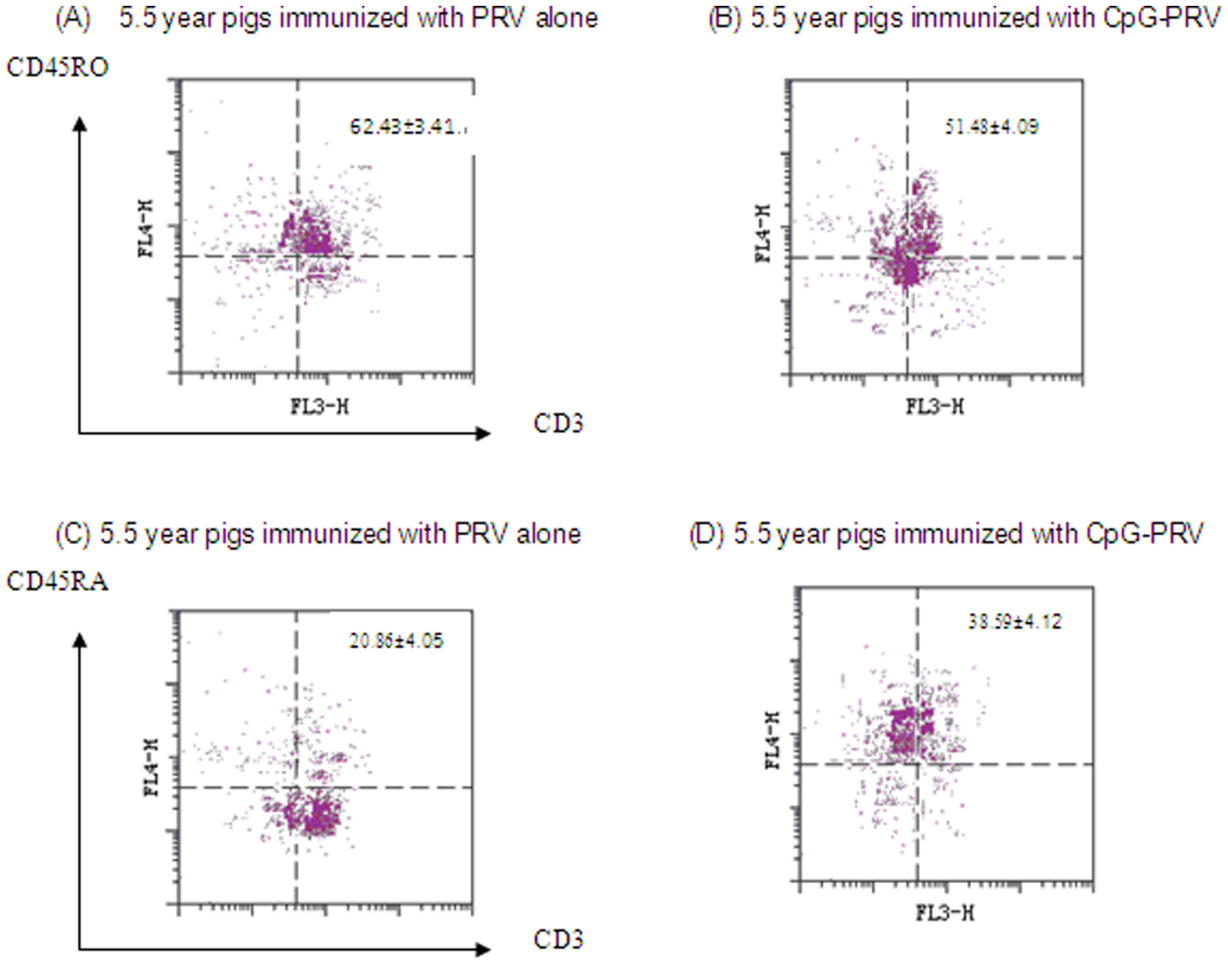
Expression of CD45RO and CD45RA in CD4+CD8+CD3+ gated lymphocytes in the 5.5 year group vaccinated with PRV-CpG vaccine or PRV alone. Legend: Shown in a representative dot plot with CD45RO or CD45RA expression plotted against CD3. The number in the upper right quadrant presents the proportion of CD45RO+ or CD45RA+ viable cells in CD4+CD8+ CD3+ lymphocytes.

To determine whether the capacity of CpG ODN restored age-related Th1 responses was due to the CpG-induced balanced CD4+, CD8+, and CD4+CD8+ T cells in aged pigs, PBMCs isolated from different age groups were used to quantify the CD4+/CD8+/CD4+CD8+ T cells. As shown in Fig. 3, there was an obvious decline in CD4+ T cells percentage in aged pigs (Fig. 3A), however, CD4+CD8+ T cells increased markedly in this age group, which was significantly higher than young group (10 wk age group) (*p*<0.05) (Fig. 3C). CpG-PRV formulation could hinder this CD4+ T cell decline and CD4+CD8+ T sharp increase in aged pigs (*p*<0.05). Since CD4+CD8+ T cells in pigs were memory T cells, we next examined the CD45RO, CD45RA phenotype on these cells from aged pigs. As expected, these cells from aged pigs (5.5 year group) were mostly positive for CD45RO, but not CD45RA (Fig. 4A, 4C), consistent with the results reported by Bailey et al [27]. Percentage of CD45RO cells decreased, while percentage of CD45RA cells enhanced in aged pigs after incorporation of CpG ODN into vaccine formulations (Fig. 4B, 4D).

**Figure 5:**
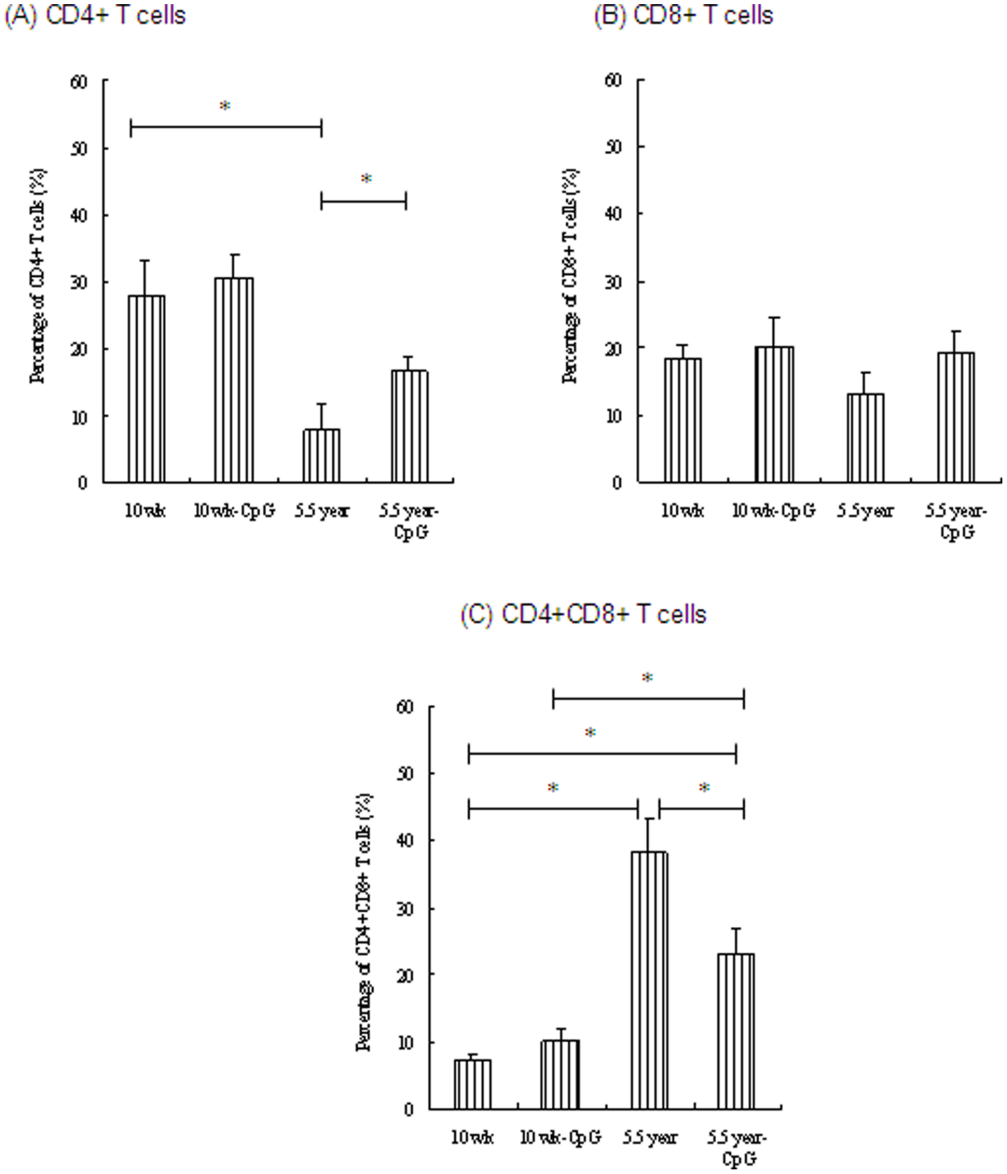
Flow cytometric characterization of CD3+ T cells ((A) CD4+ T cells, (B) CD8+ T cells, (C) CD4+CD8+ T cells) in PBMCs culture of young (10 wk) and aged (5.5 y) pigs vaccinated with PRV-CpG vaccine or PRV alone in response to PRV activation ex vivo. Legend: Samples collected 3 pools in each group were used for CD3/CD4/CD8/CD45RO and CD3/CD4/CD8/CD45RA four-color cytofluorometric analyses. Data were presented as percentage of cells positive for each surface antigen, mean percentage of leukocyte subsets +/− SEM is shown. Asterisk (*) indicates significant difference between groups (*p*<0.05).

Moreover, the CpG-restored Th1 immune responses from aged pigs might be associated with expansion change of PRV-specific T cells. To address this, PBMC cultures were incubated for 48 h with PRV, the PRV-specific T cells were detected in vitro. Unsurprisingly, PRV-specific CD4+ T cells of aged group reduced markedly compared with that of young groups (*p*<0.05) (Fig. 5A), while PRV-specific CD4+CD8+ T cells enhanced in 5.5 year age group (*p*<0.05) (Fig. 5C). Therefore, similar with the in vivo phenotype of PBMCs, CpG also could balance the occurrence of CD4+, CD4+CD8+ T cells in response to PRV antigen (CpG treatment could significantly enhance the CD4+ T cells levels in aged pigs (*p*<0.05)).

## Discussion

Aged individuals commonly exhibit deficiencies in their ability to mount protective immune responses. Attempts to immunize elderly humans or aged experimental animals against infectious agents or bacterial toxins, often resulted in failure of eliciting a protective immune response [3], [28]. It is important to pursue in an immunostimulator to improve the responsiveness of aged individuals to primary immunization. One strategy would be to develop vaccines containing suitable adjuvants to initiate and elicit of protective immunity. Our previous studies have described CpG ODN can stimulate adaptive immunity in neonatal piglets, whose immune system is functional and quantitative deficient [12], [14]. In this study, we demonstrated CpG ODN formulated with PRV vaccine could effectively restore the impaired Th1 responses in aged pigs (with good proliferative responses, PRV-specific IgG2/IgG1 ratio, and PRV-specific IFN-γ, IL-12 p40 production compared with those of young pigs), which were associated with enhanced PRV-specific CD4+ T cells priming.

Specific antibody and subtypes production, PRV-specific PBMCs proliferation and PRV-specific IFN-γ, IL-12 p40, IL-5, and IL-4 production in PBMCs, induced in different age pigs following IM inoculation of PRV vaccine formulated with or without CpG ODN, were demonstrated in this study. It had been demonstrated that Th1 cytokine IFN-γ is an important B cell switch factor for induction of antigen-specific IgG2a-secreting B cells and that many viral infections induce an antibody-mediated responses characterized by a predominance of IgG2a in mice [25], [29]. Conversely, production of antigen-specific IgG1 and IgE antibody depends at least in part, on the presence of the Th2 cytokine IL-4 [30]. In various species, measurement of certain antibody isotypes correlates directly to type 1 or type 2 T cell responses [31]. Thus, IgG2a in mice [26], IgG2 in humans [32], IgG2 in cows and IgG2 in pigs [14], [26], [31], are associated with in vivo production of IFN-γ and IL-12 p40 and can be used as correlates of type 1 responses. Indeed, our data showed that aged pigs immunized with the addition CpG ODN to PRV vaccine developed serum isotype profiles with a higher IgG2/IgG1 ratio compared with PRV-immunized aged pigs and similar IgG2/IgG1 level compared with PRV-immunized young pigs. Meanwhile good Th1 cytokines (IFN-γ, IL-12 p40) responses were also observed, all of these indicating CpG ODN could switch the immune responses from Th2-type to Th1-biased in aged pigs.

In order to analyse the effect of CpG ODN on cellular immunity, lymphocyte proliferation assays were performed. All immunised groups showed PRV-specific proliferations, which were however significantly higher in the CpG groups than in PRV alone groups (in young and aged groups respectively), and similar with the antibodies responses, CpG ODN also could restore the proliferative responses of aged pigs to the young pigs. Also in our previous study, with CpG-ODN primed animals, enhanced PRV-specific proliferation were observed [12], [14] in piglets. Recent study also showed that CpG ODNs could effectively activate antigen presenting cells (APCs), such as Dentritic cells (DCs), and facilitate antigen transport, uptake and presentation by APCs [33]. Deng et al. [34] demonstrated that decline in the type 1 T cell response in aged mice was due to age-related dysfunction of APC. So, it appears that CpG ODN has the intrinsic capacity to trigger directly or indirectly via APC the lymphocytes for better proliferation in aged pigs. This is consistent with observations of Lipford et al. [35] who demonstrated that CpG-ODN might influence the signal threshold of antigen-reactive T-cells in vivo and that this effect was due to the CpG-mediated activation of antigen-presenting cells like dendritic cells and macrophages.

Aging, is associated with an increase in the ratio of memory vs naïve T cells through both an increase in the absolute numbers of memory T cells and a reduction in the output of naïve T cells [36]. Clonal expansion in both CD4 and CD8 populations further increases the absolute numbers of memory T cells with age [37]. Our study did find that aging could decrease the total percentages of CD4+, CD8+ T cells, and increase percentages of CD4+CD8+ T cells (10 wk vs. 5.5 year) in vivo, memory T cells in pigs, but also showed a decline in the PRV-specific clonal expansion of CD4+ T cells and an increase in the PRV-specific clonal expansion of CD4+CD8+ T cells ex vivo. The addition of CpG ODN to PRV vaccine could enhance CD4+ T cells percentage and PRV-specific CD4+ T cells expansion, but reduce the CD4+CD8+ T cells and PRV-specific CD4+CD8+ T cells expansion. These data were in accord with good PRV-specific PBMCs proliferative responses and PRV-specific Th1 cytokines (IFN-γ, IL-12 p40) production in CpG-PRV immunized aged pigs. We previously found that when depleting only CD4+ T cells, but not CD4+CD8+ T cells from aged pigs, Th1 cytokine (IFN-γ) production was decreased significantly compared with non-depleted cells (data not shown). This observation mirrored the decline in PRV-specific CD4+ T cells percentage in aged pigs.

Aged host elicits decreased amount and avidity of antibody in response to various antiviral and antibacterial vaccine [38]. This is correlated with a reduced germinal center reaction and a consequent diminution in somatic hypermutation, affinity maturation [38], generation of long-lived plasma cells [39], and induction of B-cell memory [40] following immunization. Defective CD4+ T-cell help appears to play an especially important role in age-related decline of the adaptive immune responses [41]. In this regard, we demonstrated that aged pigs elicited significantly lower IgG anti-PRV responses to relative to young pigs. Meanwhile, marked reduced CD4+ T cells percentage and clone expansion were also observed in aged pigs in this study, suggesting decreased antibodies responses of aged pigs were associated with defective specific CD4+ T-cell priming. Inclusion of CpG-ODN significantly boosted CD4+ T-cell priming and restored the anti-PRV response in aged pigs. These data suggest that sufficient TLR mediated adjuvanting of PRV vaccine in aged pigs might significantly boost production of protective IgG by restoring defective CD4+ T-cell activation.

Aging, primarily due to thymic involution, is associated with an increase in the ratio of memory vs naïve T cells [36]. Clonal expansion in both CD4 and CD8 populations further increases the absolute numbers of memory T cells [37]. Our previous study found aging would decrease total percentages of CD4+, CD8+ T cells, and increase percentage of CD4+CD8+CD45RO+ T cells (10 wk vs. 5.5 year) in pigs [4]. It is well known that CpG-ODN could induce T cell activation, which would be conducted in an indirect way by antigen presentation and expression of cytokines and co-stimulatory molecules [42]. This notion is consistent with our present data which showed better CpG ODN-induecd lymphocyte proliferative responses. Meanwhile, we found that clonal expansion was particularly prominent for PRV-specific memory T cells (CD4+CD8+CD45RO+ T cells) in aged pigs, including that CpG-ODN significantly increased the CD4+CD8+CD45RA+ percentage, but decreased the CD4+CD8+CD45RO+ T cells percentage. Although it remains unclear how aging and clonal expansion affect the repertoire size and the cellular function of PRV-specific memory T cells. But at least, we believed that restored immune responses by CpG ODN in aged pigs to young pigs were correlated with observation of augmenting naive T cells by decreasing memory T cells.

In conclusion, our data suggest that an age-related Th1 response to PRV vaccine declined with aging. It appeared that these functional deficits could be overcome by treatment with CpG ODN. Although the mechanism for this functional restoration is still not completely clear, our results clearly showed that the incorporation of CpG ODN into PRV vaccine formulations, was capable of promoting development of efficient antigen specific systemic immune responses in aged pigs. We have also confirmed the ability of CpG ODN to enhance Th1 responses when used as an adjuvant for vaccination of aged animals, which were correlated with enhanced CD4+ T cells percentage and improved PRV-specific CD4+ T cells activation. While there are many publications describing the use of bacterial DNA-based vaccine strategies in young adult animals, some in aged mice, to our knowledge this is the first report of CpG ODN being used as an adjuvant for overcoming some of immune deficiencies that exist in aged pigs. Thus, our results demonstrate a utility for CpG ODN, as a safe vaccine adjuvant for promoting effective systemic immune responses in aged pig model. This agent could have important clinical uses in overcoming some of age-associated depressions in immune function that occur in response to vaccination.
